# The Ultramorphology and Sexual Dimorphism of Antennae and Sensilla in the Pale Grass Blue, *Pseudozizeeria maha* (Lepidoptera: Lycaenidae)

**DOI:** 10.3390/insects15090698

**Published:** 2024-09-14

**Authors:** Qing-Xiao Chen, Ying Han, Ya-Fei Li

**Affiliations:** Laboratory of Insect Evolution and Systematics, College of Horticulture and Plant Protection, Henan University of Science and Technology, Luoyang 471000, China; hanying220320191576@stu.haust.edu.cn (Y.H.); 15290531392@163.com (Y.-F.L.)

**Keywords:** antenna, sensillium, sexual differences, butterfly, Insecta

## Abstract

**Simple Summary:**

Antennae are prominent sensory organs in insects, facilitating various behaviors such as navigation, foraging, and pheromone communication. Due to their exclusively diurnal lifestyle, butterflies are generally thought to rely primarily on vision rather than other sensory systems, such as antennae, which have therefore received little attention. This study examined the morphological characteristics and sensilla types of the antennae in the lycaenid butterfly *Pseudozizeeria maha*, known for its sexual dimorphism in wing coloration, to determine whether there are sex-specific variations in antennal traits. While the sexes of *P. maha* have uniform clavate antennae without significant sexual differences in the overall morphology and types of sensilla, the sensilla coeloconica display significant sex-specific differences, being more abundant in females. This suggests that female *P. maha* might rely on olfactory cues for certain sex-specific behaviors, such as oviposition site selection, highlighting the importance of non-visual sensory capabilities in the life of *P. maha*.

**Abstract:**

The pale grass blue, *Pseudozizeeria maha*, is a small lycaenid butterfly widely distributed across Asia. Due to its exclusively diurnal lifestyle and conspicuous sexual dimorphism in wing coloration, vision has traditionally been regarded as the primary sensory system driving various behaviors. However, non-visual sensory systems related to sex-specific behavioral responses, such as antennae, have received far less attention. This study investigated the morphological characteristics and sensilla types of the antennae in adult *P. maha* using scanning electron microscopy, with a focus on potential sexual dimorphism. The antennae of adult *P. maha* are clavate, with no significant sexual differences in overall morphology. Six types of antennal sensilla were identified: Böhm bristles, sensilla squamiformia, sensilla trichodea, sensilla chaetica, sensilla basiconica, and sensilla coeloconica, with no sexual dimorphism observed in their morphological types or dimensions. Remarkably, the sensilla coeloconica exhibit significant sexual dimorphism, with a more prominent presence in females. This finding suggests that female *P. maha* may rely more on olfactory cues for some sex-specific behaviors, such as oviposition site selection.

## 1. Introduction

Antennae are the primary sensory organs in insects, playing essential roles in a wide range of behaviors, including navigation, foraging, oviposition site selection, and pheromone communication [1,2]. The insect antennae are abundantly equipped with sensory sensilla that can detect mechanical and chemical stimuli from the environment [3]. The antennae and their sensilla have evolved into incredibly diverse forms to meet the specific needs of different insects [4].

In Lepidoptera, the antennae exhibit significant variation, ranging from the simple filiform form found in many moth taxa [5,6,7,8,9,10,11,12,13,14,15] to the clavate form in most butterflies [16,17,18,19,20,21,22], and to the elaborate pectinate form in bombycoid moths [23,24,25]. In some cases, the moth antennae also exhibit sex-specific morphological differences, as observed in the poplar clearwing moth, *Paranthrene tabaniformis*, which possess clavate antennae in females and pectinate ones in males [26]. Given the specialized behavioral roles of antennal sensilla, such as in sex pheromone communication for courtship and in host plant selection for oviposition, significant differences in the morphological types, sizes, and distributions of sensilla have been reported both at interspecific level and between sexes in many moths [7,8,12,14,15]. However, little research has focused on butterflies, probably due to the assumption that these butterflies rely primarily on vision for their diurnal activities rather than on olfaction to the same extent as the nocturnal moths [27]. To determine whether this assumption holds true, further investigation into butterfly antennae and sensilla is necessary to explore the relationship between morphological traits and behaviors.

The pale grass blue, *Pseudozizeeria maha*, is a small-sized butterfly belonging to the second-largest butterfly family, Lycaenidae [28]. This species is widely distributed across Asia, thriving in diverse habitats such as grasslands, gardens, agricultural fields, and urban areas [29]. Its widespread distribution and adaptability to various environments suggest a need for advanced sensory capability, including vision, olfaction, thermosensation, hygrosensation, and mechanosensation. Due to conspicuous sexual dimorphism in the wing coloration of the adult *P. maha*, with males displaying light blue dorsal wings and females predominantly dark brown with occasional blue scales, it is believed that vision plays a crucial role in mate recognition [30,31]. However, the sex- and species-specific scents emitted by males may be necessary for recognizing appropriate mates and inducing receptive behavior in females at close range. Additionally, as the larvae of *P. maha* are monophagous, feeding exclusively on the leaves of *Oxalis corniculata* L. [32], it is crucial for the females to accurately perceive the physical and chemical cues of their often visually obscure host plants for oviposition. Their perception likely involves various sensilla, including those on the antennae. However, there is a significant gap in our understanding of the role of the non-visual sensory systems, such as antennae, in responding to these sex-specific behavioral demands.

In this study, we hypothesized that, in view of distinct tasks such as mate attraction and host recognition, sexual dimorphism may exist in the morphological characteristics of the antennae and sensilla in the sexually dichromatic *P. maha*. To test this hypothesis, we conducted a detailed morphological analysis of the antennae and sensilla of *P. maha* using scanning electron microscopy. Qualitative and quantitative comparisons were made to explore whether sex-specific variations in antennal traits exist in relation to different behavioral requirements.

## 2. Materials and Methods

### 2.1. Insect Collections

Adult *P. maha* were collected on the campus of Henan University of Science and Technology, Henan Province, China, in April 2023.

### 2.2. Scanning Electron Microscopy (SEM)

Live adults were anesthetized with diethyl ether. Their antennae were excised from their heads and instantly placed in a fixative solution of 2% paraformaldehyde and 2.5% glutaraldehyde in phosphate-buffered saline (PBS, 0.1 M, pH 7.2) for 24 h at 4 °C. The samples were then rinsed with PBS followed by ultrasonic cleaning. The samples were dehydrated using an ethanol concentration gradient (30%, 50%, 70%, 80%, 90%, and 95% for 10 min each, and 100% for 20 min twice) and were then transferred to the mixtures of ethanol and tertiary butanol (3:1, 1:1, and 1:3, *v*/*v*) for 15 min each, followed by 100% tertiary butanol for 30 min. The samples were freeze-dried for 2 h, adhered to the aluminum table with double-sided conductive tape, sputter-coated with gold, and imaged using a Hitachi S-3400N scanning electron microscope (Hitachi, Tokyo, Japan) at an accelerating voltage of 15 kV.

### 2.3. Terminology and Data Analysis

Terminology for antennal morphology and sensilla types followed the nomenclature presented by Schneider [1], Shields [3], and Watson et al. [33].

The SEM images at diverse magnifications, ranging from 18× to 37,000×, were used to quantify the morphological features of the antennae and sensilla in *P. maha* (9 females and 9 males). All morphometric parameters, including the lengths of antennae and dimensions of various sensilla, were measured using ImageJ 1.50i software. Mean values and standard errors were calculated with Predictive Analytics Software Statistics 20.0 (SPSS Inc., Chicago, IL, USA). Statistical significance between the sexes was assessed using the “independent samples” *t*-test in SPSS 20.0, with a threshold of *p* < 0.05.

## 3. Results

### 3.1. Antennal Morphology

**Figure 1:**
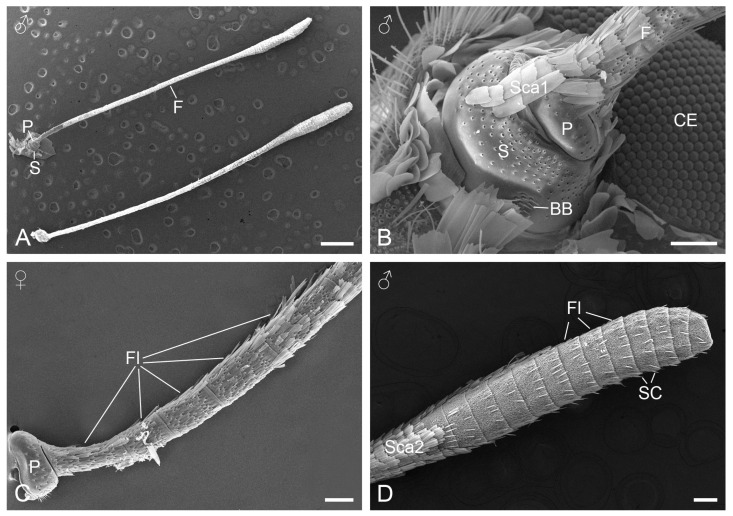
SEM micrographs of *Pseudozizeeria maha* antennae. (**A**) A pair of antennae. (**B**) Basal region of the antenna. (**C**) Pedicel and proximal flagellomeres. (**D**) Distal flagellomeres forming the antennal club. BB, Böhm’s bristle; CE, compound eye; F, flagellum; Fl, flagellomere; P, pedicel; S, scape; SC, sensillium chaeticum; Sca1, scale with pores; Sca2, scale without pores. Scale bars: (**A**) = 600 µm; (**B**,**C**) = 60 µm; (**D**) = 100 µm.

The antennae of adult *P. maha* are clavate and measure a total length of 6.45 ± 0.13 mm in females (*n* = 9) and 6.43 ± 0.14 mm in males (*n* = 9), showing no significant sexual differences. Each antenna consists of a scape, a pedicel, and a flagellum (Figure 1A). The scape is relatively enlarged and cylindrical, inserted into the head. The pedicel connects the scape to the flagellum, showing a smaller cylindrical shape than the scape (Figure 1B). The flagellum is extremely elongated and consists of 30–35 flagellomeres, which are annular and conjoined to adjacent ones, except for the apical one that is approximately pyramidal. The flagellomeres are not uniform in length: the proximal first flagellomere is longer than the second, with subsequent flagellomeres gradually lengthening towards the middle and then shortening towards the apex (Figure 1C). The apical 12 flagellomeres are wider than the preceding ones, forming the antennal club (Figure 1D).

**Figure 2:**
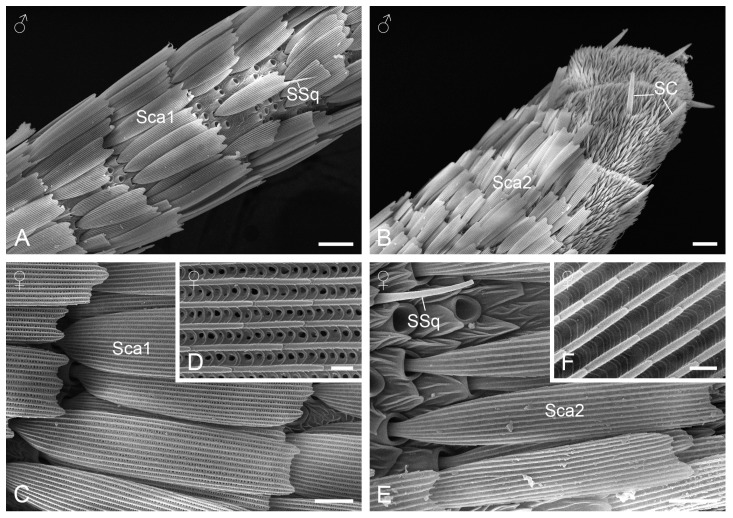
Cuticular modifications on the antennae. (**A**) Proximal flagellomeres with scales. (**B**) Dorsal view of the apical flagellomeres. (**C**) Scales with pores. (**D**) A magnified view of the subtype 1 of scale. (**E**) Scales without pores. (**F**) A magnified view of the subtype 2 of scale. SC, sensillium chaeticum; Sca1, scale with pores; Sca2, scale without pores; SSq, sensillium squamiformium. Scale bars: (**A**,**B**) = 20 µm; (**C**,**E**) = 8 µm; (**D**,**F**) = 1 µm.

In both sexes, the scape and pedicel have relatively smooth surfaces covered with overlapping flattened scales of various sizes (Figure 1B). In contrast, the surface of the flagellum is wrinkled in a reticulate pattern, with arrays of scales present on most of the proximal flagellomeres (Figure 2A). From the ventral view, the scales are absent on the apical 11 flagellomeres in females and on the apical 12 ones in males (Figure 1D). From the dorsal view, the absence of scales only occurs on the apical flagellomere in both sexes. These scaleless flagellomeres are covered with numerous tiny cuticular protrusions that form micraster-like patterns (Figure 2B).

The antennal scale consists of a basal stalk inserted into a deep socket on the cuticular surface and a main blade-shaped body that ends distally in two–three teeth (Figure 2C,D). The scale body is ornamented with arrays of spaced longitudinal ridges, which are joined at intervals by short cross-ribs. Along these longitudinal ridges is a series of flanges that angle up along the ridges, forming ridge-lamellae. Two distinct subtypes of scales can be distinguished: one subtype (Sca1) features a tiny pore on the rectangular flat formed by the longitudinal ridges and cross-ribs, mainly distributed on the proximal portion of the antenna (Figure 2C, inset). The other subtype (Sca2) lacks pores on all flats and appears on the distal portion of the flagellum (Figure 2D, inset).

### 3.2. Sensilla on the Antennae

The antennae of both sexes contain six types of sensilla: Böhm bristles, sensilla squamiformia, sensilla trichodea, sensilla chaetica, sensilla basiconica, and sensilla coeloconica.

#### 3.2.1. Böhm’s Bristles

**Figure 3:**
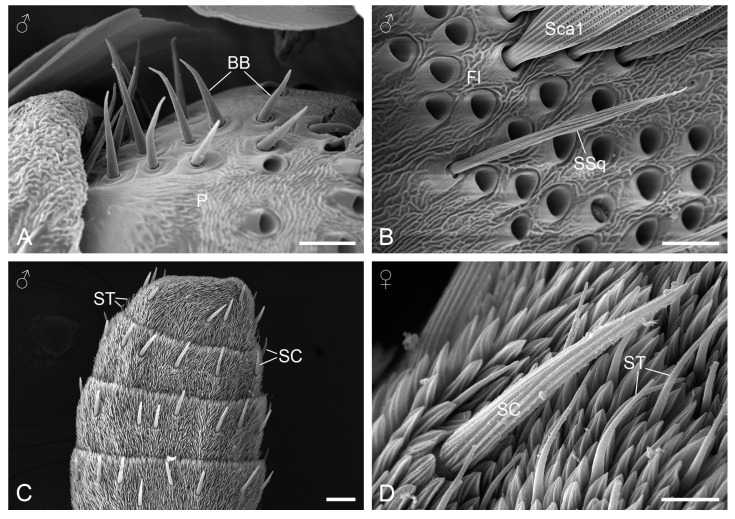
Antennal sensilla. (**A**) Böhm’s bristles on the pedicel. (**B**) Sensillium squamiformium on a flagellomere with scales. (**C**) Rows of sensilla chaetica on the scaleless flagellomeres. (**D**) Sensillium chaeticum. BB, Böhm’s bristle; Fl, flagellomere; P, pedicel; SC, sensillium chaeticum; Sca1, scale with pores; SSq, sensillium squamiformium; ST, sensillium trichodeum. Scale bars: (**A**,**B**,**D**) = 8 µm; (**C**) = 40 µm.

Böhm’s bristles are arranged in two discrete clusters: one on the ventral side of the scape and the other on the dorsal side of the pedicel (Figure 1B). This arrangement is consistent in both sexes. Each cluster is populated with 17 to 20 sensory bristles, which are characterized by sharp tips and smooth surfaces emerging from the sunken sockets in the antennal cuticle (Figure 3A). There are no statistically significant differences in the mean lengths and basal widths of the scapal and pedicallar bristles between the sexes (Table 1).

#### 3.2.2. Sensilla Squamiformia

Sensilla squamiformia are scattered across the flagellomeres covered with scales, interspersed randomly among the scales. The sensilla are spine-shaped and inserted into deep sockets. They have a scale-like appearance ornamented with arrays of longitudinal ridges, ridge-lamellae, and short cross-ribs (Figure 3B). The number of sensilla squamiformia is difficult to determine accurately because they are always covered by scales. The sensilla do not differ significantly in length and basal width between the sexes (Table 1).

#### 3.2.3. Sensilla Chaetica

Sensilla chaetica are located on the scaleless flagellomeres. They are arranged in a row along the ventral surface and lateral edges of each scaleless flagellomere (Figure 3C), with the exception of the apical flagellomere, which has 2–3 rows. The sensilla chaetica assume straight bristles with blunt tips and inserted into circular membranes at the base, inclined at a 30–40° angle toward the antennal apex. These bristles possess longitudinal ridges with ridge-lamellae on their surfaces (Figure 3D), similar to sensilla squamiformia but lacking short cross-ribs between the ridges. At more than 4000× magnifications, no evidence of extensive wall pores is visible on the surface, though a single pore may or may not be present at the tip. No significant differences are observed in the length and basal width between the sexes (Table 1).

#### 3.2.4. Sensilla Trichodea

**Figure 4:**
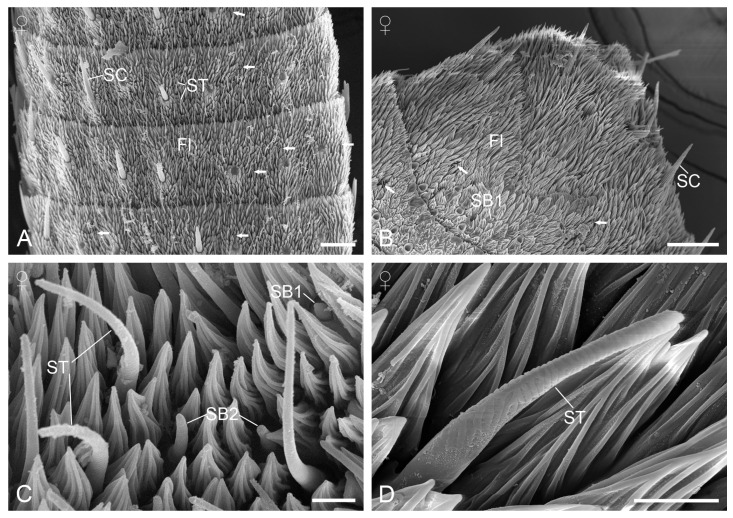
Sensilla on the antennal club. (**A**) Ventral view of the fifth to eighth apical flagellomeres, showing numerous sensilla trichodea and sensilla coeloconica (arrows). (**B**) Dorsal view of the apical flagellomeres. Arrows indicate sensilla coeloconica. (**C**) Sensilla trichodea and basiconica. (**D**) Sensillium trichodeum. Fl, flagellomere; SB1, sensillium basiconicum 1; SB2, sensillium basiconicum 2; SC, sensillium chaeticum; ST, sensillium trichodeum. Scale bars: (**A**,**B**) = 30 µm; (**C**,**D**) = 3 µm.

Sensilla trichodea are the most abundant type of sensilla on the scaleless flagellomeres, with their numbers being significantly greater on the ventral surface than on the dorsal surface (Figure 4A,B). They are interspersed among the cuticular micrasters and have cuticular sockets, which are difficult to observe due to obstruction by other sensilla and micrasters (Figure 4C). The sensilla are slender and slightly curved toward the antennal tip, with a helical texture and tiny wall pores. The pores are unevenly spaced along the helical grooves, with fewer pores near the base than toward the tip (Figure 4D). No significant differences are observed in the length and basal width of sensilla trichodea between the sexes (Table 1).

#### 3.2.5. Sensilla Coeloconica

**Figure 5:**
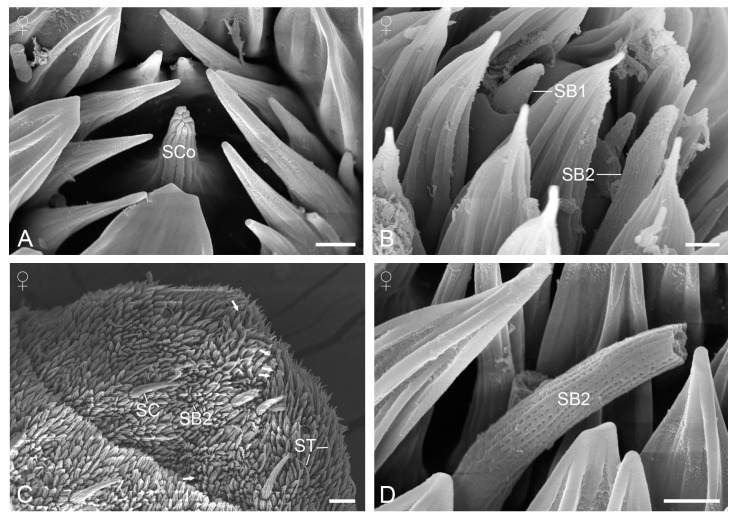
Sensilla coeloconica and basiconica. (**A**) Sensillium coeloconicum. (**B**) Two subtypes of sensilla basiconica. (**C**) Ventral view of the apical flagellomere. Arrows indicate sensilla basiconica 1. (**D**) Sensillium basiconicum 2. SB1, sensillium basiconicum 1; SB2, sensillium basiconicum 2; SC, sensillium chaeticum; SCo, sensillium coeloconicum; ST, sensillium styloconicum. Scale bars: (**A**,**B**,**D**) = 1 µm; (**C**) = 10 μm.

Sensilla coeloconica are located on the scaleless regions of the distal flagellomeres (Figure 4A,B). Each sensillum consists of a pit encircled by 11–14 cuticular spines that converge towards the center and a sensory peg emerging from the center of the pit. The central peg has prominent longitudinal ridges with wall pores visible within the grooves (Figure 5A). The sensilla exhibit sex-specific differences in number and distribution. In females, approximately 18 sensilla coeloconica are present on the ventral surface, with 1–3 on one or both sides of the first 12 flagellomeres (Figure 4A), and about 10 on the dorsal surface, with 1–2 on each side of the second, third, and fourth flagellomeres (Figure 4B). In males, only a single sensillum is found on the dorsal surface of the second and third flagellomeres, respectively, and one on the ventral surface of the first flagellomere. The dimensions of the sensilla are difficult to measure reliably because they are often covered up by surrounding cuticular micrasters.

#### 3.2.6. Sensilla Basiconica

Sensilla basiconica are present on the scaleless flagellomeres and can be classified into two subtypes based on distinct morphological features. Subtype 1 appears as a stout sensory cone on a raised socket, with the cone tip barely extending above the surrounding cuticular micrasters (Figure 5B). Subtype 1 sensilla are located on the dorsal and ventral surfaces of the first two flagellomeres, with a greater aggregation observed on the ventral surface of the apical flagellomere (Figure 5C). Subtype 2 is hair-like with a blunt tip, significantly shorter than the sensilla trichodea but slightly longer than the cuticular micrasters (Figure 4C). They are characterized by longitudinal ridges with wall pores arranged in rows within shallow furrows (Figure 5D). Subtype 2 sensilla are more numerous than subtype 1 and are scattered across the scaleless flagellomeres, both ventrally and dorsally. Neither subtype of sensilla basiconica shows significant differences in length and basal width between the sexes (Table 1).

## 4. Discussion

Based on our investigations, the antennae of adult *P. maha* are clavate in form, with no significant sexual dimorphism. Considering that insect antennae are known to contribute to stability and orientation during flight [34], the absence of sexual dimorphism in the overall antennal morphology suggests that both sexes might have similar flight capabilities. This uniformity may be essential for navigating diverse habitats such as grasslands, gardens, agricultural fields, and urban areas. Extending further to the entire butterfly Papilionoidea, the clavate shape of antennae has been documented in another lycaenid species, *Chilades pandava* [19], as well as in some species of Nymphalidae [16,18,21,22] and Pieridae [17,20]. In contrast to the filiform antennae found in the phylogenetically ancient lepidopteran family Micropterygidae [5] and in other nocturnal moth families [8,10,12,13], the clavate antennae of diurnal butterflies may provide an evolutionary advantage in terms of enhancing complex flight strategies, including the precise detection of airflow and body position for navigation, foraging, and the avoidance of obstacles and predators.

The antennal sensilla of the lycaenid *P. maha* exhibit hardly any sexual dimorphism in their morphological types or dimensions, suggesting a conserved functional design between the sexes. An exception to this is the sensilla coeloconica, which exhibit sex-specific differences in number and distribution, with a more prominent presence in females. In Lepidoptera, the sensilla coeloconica on the antennae can vary greatly in number, ranging from being unreported in two pierid butterflies, *Colia eurytheme* and *C. philodice* [17], to as many as 600–800 per antenna in the silkworm, *Bombyx mori* [23]. The sensilla coeloconica in *P. maha* are morphologically similar to those found in *B. mori* [23] and in some butterflies from the *Pieridae* [20], *Nymphalidae* [21,22], and *Hesperiidae* [35]. However, they differ from those observed in another lycaenid, *C. pandava*, where they are arranged in clusters of nine on a circular tubercle at the apical flagellomere [19]. Given that the sensilla coeloconica are chemo-, thermo-, and hydrosensitive [3], these variations in number and morphology are likely related to ecological and behavioral needs. Pophof [36] considered that the sensilla coeloconica might be involved in the selection of oviposition sites in *B. mori*. Thus, the sexual differences in the *P. maha* sensilla coeloconica may indicate that the females are more reliant on olfactory cues provided by these sensilla to find suitable oviposition sites. However, due to the limitations of SEM in observing sensilla coeloconica, such as their small size and potential obstruction by other structures, a thorough ultrastructural examination using transmission electron microscopy will be essential for a detailed and accurate analysis of these sensilla in future studies.

In addition to sensilla, the antennae of *P. maha* exhibit complex cuticular modifications, with overlapping scales present on most of the proximal flagellomeres and micraster-like protrusions on the distal scaleless ones. These cuticular scales and micrasters increase antennal surface roughness to resist droplets and mist conditions, thereby reducing the risk of antennal contamination by microbial pathogens and particles [33]. Similar cuticular modifications have been identified in the lycaenid *C. pandava* [19] and other butterflies [16,17,18,20,21,22,35], indicating a well-conserved antennal design among butterflies. Despite the similarity in modifications, the antennal surfaces exhibit distinct topographies among different butterfly taxa. One type features elliptical depressions (i.e., sulci, one or more per flagellomere), a characteristic commonly found in Pieridae [17,20]. Another type features three longitudinal ridges delimiting two shallow grooves, observed in some Nymphalidae butterflies [16,18]. Additionally, an intermediate type with a ridge and a double row of depressions is also present in certain Nymphalidae species [22,37]. These grooves and depressions are always filled with a high density of sensilla trichodea, indicating that these areas might have increased sensitivity to specific olfactory signals. In contrast, none of the aforementioned cuticular topographies are present on the antennae of the lycaenid *P. maha* or *C. pandava* [19] as well as Hesperiidae species [35], nor is there any accumulation of sensilla trichodea. Whether these varied topographies have any physiological or taxonomic significance requires further investigation.

Previous research on antennal characteristics has mainly focused on moths, particularly important pest species [7,10,11,12,13], while diurnal butterflies have received much less attention. This is likely due to the traditional view that diurnal butterflies rely more on vision than on olfaction [27]. However, our study demonstrated that, despite minimal sexual dimorphism in the antennae and sensilla of *P. maha*, the distinct sexual differences in sensilla coeloconica might support our hypothesis that sex-specific behaviors, such as oviposition site selection by females, could be reflected in the morphological characteristics of butterfly antennae and sensilla. These findings highlight the need for further research on butterfly antennae and sensilla to better understand their non-visual sensory capabilities and to provide valuable insights for taxonomic and phylogenetic analyses.

## Figures and Tables

**Table 1:** Dimensions of antennal sensilla of female and male *P. maha*.

Sensilla Types/Subtypes	Length (μm)			Basal Width (μm)		
	Female	Male	*t*-Test	Female	Male	*t*-Test
Böhm’s bristles on scape	13.75 ± 0.38 (11)	14.46 ± 0.42 (11)	NS	2.13 ± 0.04 (14)	2.12 ± 0.07 (14)	NS
Böhm’s bristles on pedicel	14.07 ± 0.42 (8)	13.37 ± 0.43 (8)	NS	2.01 ± 0.04 (10)	1.99 ± 0.08 (10)	NS
Sensilla squamiformia	32.65 ± 0.59 (7)	33.52 ± 0.96 (7)	NS	1.76 ± 0.02 (7)	1.84 ± 0.05 (7)	NS
Sensilla chaetica	33.54 ± 2.25 (8)	32.62 ± 1.57 (8)	NS	5.13 ± 0.15 (8)	5.35 ± 0.14 (8)	NS
Sensilla trichodea	13.73 ± 0.49 (7)	16.69 ± 1.46 (7)	NS	1.53 ± 0.03 (7)	1.55 ± 0.06 (7)	NS
Sensilla basiconica 1	2.49 ± 0.35 (4)	2.55 ± 0.11 (4)	NS	1.46 ± 0.19 (4)	1.11 ± 0.04 (4)	NS
Sensilla basiconica 2	6.43 ± 0.70 (7)	6.82 ± 0.57 (7)	NS	1.08 ± 0.05 (8)	1.08 ± 0.06 (8)	NS
Sensilla coeloconica	2.34 ± 0.17 (4)	−	−	1.15 ± 0.03 (4)	−	−

Data are presented as mean ± SE (*n*). *n*, sample size. − indicates that there was an insufficient number of sensilla for the analysis. NS: not significant (*p* ≥ 0.05) in *t*-test.

## Data Availability

The data presented in this study are available on request from the corresponding author.
